# Possible advantages of early stabilization of spinal fractures in multiply injured patients with leading thoracic trauma - analysis based on the TraumaRegister DGU®

**DOI:** 10.1186/s13049-020-00737-6

**Published:** 2020-05-24

**Authors:** Sven Hager, Helge Eberbach, Rolf Lefering, Thorsten O. Hammer, David Kubosch, Christoph Jäger, Norbert P. Südkamp, Jörg Bayer, TraumaRegister DGU®

**Affiliations:** 1 Department of Surgery, Bautzen Hospital, Oberlausitz-Kliniken gGmbH, Am Stadtwall 3, 02625 Bautzen, Germany; 2 Department of Orthopedics and Trauma Surgery, Medical Center – Albert-Ludwigs-University of Freiburg, Faculty of Medicine, Hugstetter Str. 55, 79106 Freiburg, Germany; 3 IFOM - Institute for Research in Operative Medicine, University Witten/Herdecke, Faculty of Health, Ostmerheimer Str. 200, 51109 Köln, Germany; 4 Department of Anesthesiology and Critical Care, Medical Center – Albert-Ludwigs-University of Freiburg, Faculty of Medicine, Hugstetter Str. 55, 79106 Freiburg, Germany; 5 Committee on Emergency Medicine, Intensive Care and Trauma Management of the German Trauma Society (Sektion NIS), Berlin, Germany

**Keywords:** Spinal fracture, Thoracic trauma, Multiply injured patients, Lung failure, Multiple organ failure

## Abstract

**Background:**

Major trauma often comprises fractures of the thoracolumbar spine and these are often accompanied by relevant thoracic trauma. Major complications can be ascribed to substantial simultaneous trauma to the chest and concomitant immobilization due to spinal instability, pain or neurological dysfunction, impairing the respiratory system individually and together. Thus, we proposed that an early stabilization of thoracolumbar spine fractures will result in significant benefits regarding respiratory organ function, multiple organ failure and length of ICU / hospital stay.

**Methods:**

Patients documented in the TraumaRegister DGU®, aged ≥16 years, ISS ≥ 16, AIS_Thorax_ ≥ 3 with a concomitant thoracic and / or lumbar spine injury severity (AIS_Spine_) ≥ 3 were analyzed. Penetrating injuries and severe injuries to head, abdomen or extremities (AIS ≥ 3) led to patient exclusion. Groups with fractures of the lumbar (LS) or thoracic spine (TS) were formed according to the severity of spinal trauma (AIS_spine_): AIS_LS_ = 3, AIS_LS_ = 4–5, AIS_TS_ = 3 and AIS_TS_ = 4–5, respectively.

**Results:**

1740 patients remained for analysis, with 1338 (76.9%) undergoing spinal surgery within their hospital stay. 976 (72.9%) had spine surgery within the first 72 h, 362 (27.1%) later on. Patients with injuries to the thoracic spine (AIS_TS_ = 3) or lumbar spine (AIS_LS_ = 3) significantly benefit from early surgical intervention concerning ventilation time (AIS_LS_ = 3 only), ARDS, multiple organ failure, sepsis rate (AIS_TS_ = 3 only), length of stay in the intensive care unit and length of hospital stay. In multiple injured patients with at least severe thoracic spine trauma (AIS_TS_ ≥ 4) early surgery showed a significantly shorter ventilation time, decreased sepsis rate as well as shorter time spend in the ICU and in hospital.

**Conclusions:**

Multiply injured patients with at least serious thoracic trauma (AIS_Thorax_ ≥ 3) and accompanying spine trauma can significantly benefit from early spine stabilization within the first 72 h after hospital admission. Based on the presented data, primary spine surgery within 72 h for fracture stabilization in multiply injured patients with leading thoracic trauma, especially in patients suffering from fractures of the thoracic spine, seems to be beneficial.

## Background

There is an ongoing discussion in the current literature on the optimal timing of stabilization for thoracolumbar spine fractures in multiply injured patients (MIP). Spinal injuries are relevant in MIP since one third suffer from these injuries after experiencing major trauma [1–4].

The anatomical distribution of spinal fractures is around 48% at the lumbar, 31% at the thoracic and 21% at the cervical spine level [5]. The main blunt trauma mechanisms are traffic accidents and accidental or suicidal falls [2, 5] which transmit enough energy to the trunk and its adjacent organ systems to cause spinal injury. Consecutively, patients with thoracic spine or upper lumbar spine trauma very likely suffer from concomitant relevant thoracic trauma, respectively rip fractures (30%), lung contusion (30–64%), pneumothorax (24–26%) and pleural effusion / hemothorax (39%) - altogether conditions often associated with significant impairment of the respiratory system [6–8]. Concerning the lower lumbar spine, injuries to that region commonly involve pelvic (25%) and abdominal trauma (30–51%) and consecutive bleeding [9–11].

Major consequences related to thoracolumbar spine injuries can be ascribed to substantial trauma to the chest and concomitant immobilization due to spinal instability, pain or neurological dysfunction, respectively, which altogether evidently affect the respiratory system [6, 12]. Especially in patients with leading thoracic trauma this injury itself very often mandates emergency procedures, intensive care therapy and is highly associated with respiratory failure and multiple organ dysfunction syndrome [13, 14]. Consecutively, MIP with leading thoracic trauma and thoracolumbar spine fractures are at even higher risk for respiratory complications.

Among MIP with an ISS ≥ 16 there is some evidence of beneficial spinal stabilization within 72 h after hospital admission regarding the length of stay on intensive care unit (ICU) or in hospital, fewer days of mechanical ventilation and lower rates of sepsis [1, 15–19]. Yet, these studies consist of patients with different leading injuries (e.g. severe head injury, or severe injury to the extremities) which inherently influence the associated stays on intensive care units, mandatory ventilation and risk for sepsis and multiple organ dysfunction.

As mentioned above, the combination of relevant thoracic and thoracolumbar spine injury implies the risk of respiratory deterioration or aggravation of organ dysfunction. Yet, for the specific subgroup of MIP with leading thoracic trauma there is no data about the optimal timing of thoracolumbar spine fracture stabilization. Elucidating this topic seems relevant, since the dilemma of finding the best time to operate on an already respiratory impaired patient – especially suffering from spinal trauma without neurologic deficits – is evident.

This is why we conducted this study and proposed that an early stabilization of thoracolumbar spine fractures will result in significant benefits regarding organ function and length of ICU / hospital stay.

## Methods

### Database

The TraumaRegister DGU® of the German Trauma Society (Deutsche Gesellschaft für Unfallchirurgie, DGU) was founded in 1993. The aim of this multi-centre database is a pseudonymized and standardized documentation of severely injured patients.

Data are collected prospectively in four consecutive time phases from the site of the accident until discharge from hospital: A) Pre-hospital phase, B) Emergency room and initial surgery, C) Intensive care unit and D) Discharge. The documentation includes detailed information on demographics, injury pattern, comorbidities, pre- and in-hospital management, course on intensive care unit, relevant laboratory findings including data on transfusion and outcome of each individual. The inclusion criterion is admission to hospital via the emergency room with subsequent ICU / IMC care, including patients who reach the hospital with vital signs and die before admission to ICU.

The infrastructure for documentation, data management, and data analysis is provided by AUC - Academy for Trauma Surgery (AUC - Akademie der Unfallchirurgie GmbH), a company affiliated to the German Trauma Society. The scientific leadership is provided by the Committee on Emergency Medicine, Intensive Care and Trauma Management (Sektion NIS) of the German Trauma Society. The participating hospitals submit their data pseudonymized into a central database via a web-based application. Scientific data analysis is approved according to a peer review procedure laid down in the publication guideline of TraumaRegister DGU®.

The participating hospitals are primarily located in Germany (90%), but a rising number of hospitals of other countries contribute data as well (at the moment from Austria, Belgium, China, Finland, Luxem-bourg, Slovenia, Switzerland, The Netherlands, and the United Arab Emirates). Currently, approx. 35,000 cases from almost 700 hospitals are entered into the database per year.

Participation in TraumaRegister DGU® is voluntary. For hospitals associated with TraumaNetzwerk DGU®, however, the entry of at least a basic data set is obligatory for reasons of quality assurance.

### Patient selection

Patients documented between 2009 and 2015 in the TR-DGU were analyzed for eligibility. Patient selection was carried out according to the following criteria:

#### Inclusion criteria

(1) European trauma centers, (2) age ≥ 16 years, (3) ISS ≥ 16, (4) chest injury severity (AIS_Thorax_) ≥ 3 and (5) an additional thoracic and / or lumbar spine injury with severity (AIS_Spine_) ≥ 3.

#### Exclusion criteria

(1) patients with a basic dataset only (reduced dataset without interventions), (2) patients transferred to a referring hospital longer than 24 h after trauma (3) patients transferred into another institution within 2 days after admission (because of lack of outcome data) (4) penetrating injury, (5) severe injury to either the head, abdomen or extremities defined as AIS_Head_ > 3, AIS_Abdomen_ > 3 and AIS_Extremity_ > 3.

The selected patients were divided into two interventional subgroups regarding the timing of surgery. The assignment to either subgroup was based on the time of the first spine operation, irrespective of the surgical approach for spinal stabilization:

Early spinal stabilization was defined as surgery within the first 72 h after hospital admission (early surgical intervention = ESI) and late stabilization was defined as surgery more than 72 h after admission (late surgical intervention = LSI). These time periods were chosen in accordance with previously published studies [1, 19–22].

Additionally, for reasons of comparison, patients without surgical intervention (no surgical intervention = NSI) are stated, also.

Another differentiation was made according to the localization and severity of the spinal injury: MIP with thoracic trauma and fractures of the lumbar spine (LS) or thoracic spine (TS) were distributed according to the severity of spinal trauma (AIS_spine_) into the following groups: AIS_LS_ = 3, AIS_LS_ = 4–5, AIS_TS_ = 3 and AIS_TS_ = 4–5, respectively.

Injuries were graded according to the 2005 / update 2008 version of the Abbreviated Injury Scale (AIS) [23], and the Injury Severity Score (ISS) was calculated to describe the overall injury severity [24].

### Statistical analysis

Statistical analysis evaluated the demographic and clinical characteristics comparing the aforementioned groups. Continuous variables are presented as mean with standard deviation (SD), while categorical variables are presented as number of cases with percentages. For each variable the statistics refer to patients with valid data sets only, therefore, the total number of patients analyzed may vary marginally.

The treatment groups were compared using chi-squared test in case of a categorical variable, and the non-parametric Kruskal-Wallis test in case of continuous measurements, or the Mann Whitney U-Test. Pairwise comparisons (in case of a significant overall test) were calculated for selected variables only, in order to limit the number of test statistics. *P*-values ≤0.05 were regarded as statistically significant, but should be interpreted carefully in this explorative analysis.

Subgroups of AIS_TS_ and AIS_LS_ were analyzed concerning clinical outcome parameters. The outcome parameters included were: Ventilation time (VT), length of ICU stay (ICUT), length of hospital stay (HT), multi organ failure (MOF), sepsis rate (SR), mortality, blood transfusion, neurologic outcome according to the Glasgow Outcome Scale (GOS). For these analyses, early (within 24 h of hospital admission) deceased patients were excluded.

All data were analyzed using SPSS, version 22.0 (IBM Inc., Armonk, NY, USA).

The present study is in line with the publication guidelines of the TR-DGU and registered as TR-DGU project ID 2016–002.

## Results

### Demographics

The initial database contained 145,518 patients from the study period. After applying the inclusion and exclusion criteria, a total of 1740 patients remained for analysis. Of these patients, 1338 (76.9%) had spinal surgery within their hospital stay, 976 of these (72.9%) within the first 72 h and 362 (27.1%) later on. The majority of patients (88.6%) were treated at a supra-regional level I trauma center, 10.3% at a regional level II center and 1.1% at a local level III trauma center. The following European countries contributed the stated percentages of patients to our finally analyzed cohort: Germany (77.1%), Austria (9.4%), the Netherlands (6.4%), Switzerland (4.4%), Finland (1.5%), Belgium (0.8%) and Slovenia (0.4%). Further demographic characteristics are listed in Table 1.

**Table 1:** Demographics of multiply injured patients with severe thoracic trauma and concomitant spine trauma

	Total	No Surgery (NSI) *n* = 402	Early Surgery (ESI) *n* = 976	Late Surgery (LSI) *n* = 362	*P*-value (ESI versus LSI)
Male gender​	76.2 (1320)	76.5 (306)​	76.2 (739) ​	76.0 (275)​	0.98
Mean age	48.0 (SD 18.7)	51.2 (SD 20.1)	45.9 (SD 17.6)	50.1 (SD 19.1)	< 0.001
Mean ISS	26.6 (SD 9.6)	26.5 (SD 10.6)​	27.5 (SD 9.3)	25.6 (SD 8.9)	0.001
ISS ≥ 25	59.8 (1041)	53.2 (214)	65.3 (637)	52.2 (190)	< 0.001
RR_sys_ ≤ 90 mmHg prehospital	16.2 (214)	22.2 (66)	13.4 (99)	17.0 (49)	0.002
RR_sys_ ≤ 90 mmHg on admission	13.4 (218)	16.7 (60)	11.8 (108)	14.6 (50)	0.051
Need for blood transfusion	17.2 (299)	15.8 (63)	18.3 (179)	15.7 (57)	0.36
Need for thoracic drain prehospital	5.4 (78)	6.6 (22)	4.9 (39)	5.6 (17)	0.52
Need for thoracic drain trauma room	26.7 (456)​	29.7 (115)	26.3 (255)	24.3 (86)	0.23

Percentages (number of patients) and mean values (SD, standard deviation) are given. The *p*-values are derived comparing the ESI and LSI group. The NSI group is listed to display the complete dataset

While a considerable percentage of patients in all subgroups (NSI, ESI, LSI) suffered from prehospital hypotension (RR_sys_ ≤ 90 mmHg), initial prehospital treatment reduced the prevalence of shock at the time of hospital admission in all subgroups. At this point in time no significant statistical differences existed regarding the percentage of patients in shock at hospital admission, although there was a tendency towards a higher percentage of instable patients in the late surgery group (LSI) compared to the ESI (Table 1).

While patients in the LSI group had a significantly higher mean age than patients in the ESI group (*p* < 0.001), patients in the later group had a significantly higher mean ISS (*p* = 0.001) and higher percentage of patients with an ISS ≥ 25 (*p* < 0.001) (Table 1).

We also analyzed the need for blood transfusion, prehospital or / and trauma room tube thoracostomy as emergency procedures and found no statistical differences regarding these interventions in our subgroups ESI versus LSI (Table 1).

According to the trauma severity the vast majority of patients in our collective was admitted to the ICU (NSI: 84.3% (*n* = 339); ESI: 97.6% (*n* = 953); LSI: 93.6% (*n* = 339)).

### Mortality

The early mortality (death within 24 h after hospital admission) was highest in the NSI group, as was the overall hospital mortality. More patients died during their hospital stay in the ESI group, compared to the LSI (3.5% vs. 1.9%) (Table 2).

**Table 2:** Observed and predicted mortality for different surgical strategies

	Total	No Surgery (NSI)	Early Surgery (ESI)	Late Surgery (LSI)
Mortality within the first 24 h	2.2% (39)	9.0% (36)	0.3% (3)	(none)
Mortality during hospital stay	6.5% (113)	17.9% (72)	3.5% (34)	1.9% (7)
RISC II prognosis	8.3%	14.0%	5.9%	8.2%

Mortality rate (number of patients) and calculated predicted mortality (RISC II) are given for the according subgroups

Calculated predicted mortality was assessed by the RISC II score [25] and was higher in the ESI and LSI group compared to the actually observed mortality in the respective subgroups (Table 2).

Patients with fractures of the thoracic spine had a significantly higher mortality compared to patients with fractures of the lumbar spine (8.3% vs. 3.2%, *p* < 0.001).

Because of overlapping data of 85 patients who sustained fractures to both the lumbar and the thoracic spine, 5 deaths in the NSI group and 2 deaths in the ESI group appear in both fractions.

### Surgical therapy

An early surgical intervention (ESI) was performed more often in all MIP, irrespective of the spine fracture level. Additionally, with higher AIS_TS_ or AIS_LS_ the percentage of patients receiving an early surgical intervention increased. Detailed data are shown in Table 3.

**Table 3:** Distribution of surgical strategies according to different spine trauma locations and severity

	Total	No Surgery (NSI)	Early Surgery (ESI)	Late Surgery (LSI)
**Fracture of the thoracic spine AIS ≥ 3**	69.1 (1188)	24.8 (295)	54.7 (650)	20.5 (243)
AIS TS = 3	51.8 (685)	29.2 (200)	43.2 (296)	27.6 (189)
AIS TS = 4	14.8 (61)	23.0 (14)	59.0 (36)	18.0 (11)
AIS TS = 5	33.4 (442)	18.3 (81)	71.9 (318)	9.7 (43)
**Fracture of the lumbar spine AIS ≥ 3**	37.6 (645)	22.5 (145)	55.8 (360)	21.7 (140)
AIS LS = 3	75.0 (484)	24.6 (119)	49.4 (239)	46.0 (126)
AIS LS = 4	9.6 (62)	17.7 (11)	74.2 (46)	8.1 (5)
AIS LS = 5	15.4 (99)	15.2 (15)	75.8 (75)	9.1 (9)

Surgical timing in correlation to the severity of the concomitant thoracic or lumbar spine fracture. Percentages (number of patients) are given

To better analyze the effect of spinal trauma severity in the context of thoracic trauma and best surgical timing we devided our fracture sites (thoracic, lumbar) in AIS groups “3” (AIS_spine_ = 3) and “4/5” (AIS_spine_ = 4 and 5), respectively.

For further analysis, early (within 24 h of hospital admission) deceased patients were excluded from the following tables.

### Thoracic spine (TS); AIS_TS_ = 3

Within the subgroup of MIP with serious fractures of the thoracic spine (AIS_TS_ = 3) ESI treatment resulted in significant reduction of multiple organ failure (MOF), ARDS (lung failure) and sepsis rate (SR). Furthermore, the length of stay in the intensive care unit (ICUT) and in hospital (HT) was significantly longer in the LSI group. The blood transfusion rate did not differ significantly among the subgroups as well as the ventilation time (VT). The comprehensive data is shown in Table 4.

**Table 4:** Outcome of multiple injured patients with severe thoracic trauma and concomitant serious thoracic spine trauma (AIS_TS_ = 3)

	No Surgery (NSI) *n* = 180	Early Surgery (ESI) *n* = 295	Late Surgery (LSI) *n* = 189	*P*-value (ESI versus LSI)
Mean ISS	22.0	20.3	21.8	0.12
Days on ventilator	5.1 [1]	5.6 [1]	8.4 [1]	0.21
Days on ICU	9.0 [5]	10.4 [6]	14.5 [9]	0.004
Days in hospital	20.6 [17]	23.9 [20]	31.0 [26]	< 0.001
MOF	26.3 (40)	20.4 (56)	39.0 (67)	< 0.001
Lung failure (ARDS)	24.3 (37)	21.2 (58)	30.2 (52)	0.031
Sepsis	9.7 (14)	6.8 (18)	12.4 (21)	0.045
Blood transfusion	11.7 (21)	9.8 (29)	13.2 (25)	0.25
GOS 3	8.2 (14)	5.1 (15)	12.2 (23)	0.003
GOS 4	22.4 (38)	22.3 (65)	31.2 (59)	
GOS 5	55.3 (94)	68.5 (200)	54.5 (103)	

For VT, ICUT and HT the mean [median] is listed. For MOF; lung failure; sepsis, blood transfusion and GOS percentages (patient numbers) are given. The *p*-values are derived comparing the ESI and LSI group. The NSI group is listed to display the complete dataset

Concerning the neurological outcome, measured by the Glasgow outcome scale (GOS) at hospital discharge, MIP with relevant thoracic trauma and serious thoracic spine injury showed significantly better GOS scores (improved neurologic recovery / function) at discharge in the ESI group, compared to LSI (Table 4).

To check for a possible influence of the thoracic trauma severity on the timing of surgery, the distribution of AIS_Thorax_ severity was assessed in the according subgroups and was statistically not significant (ESI vs. LSI, *p* = 0.21; Table 5). Additionally, the mean ISS did not differ significantly among the subgroups.

**Table 5:** Distribution of thoracic injury severity in multiply injured patients suffering from serious thoracic spine injury

	No Surgery (NSI) *n* = 180	Early Surgery (ESI) *n* = 295	Late Surgery (LSI) *n* = 189
AIS _Thorax_ = 3	67.2 (121)	62.7 (185)	63.0 (119)
AIS _Thorax_ = 4	18.3 (33)	25.1 (74)	20.1 (38)
AIS _Thorax_ = 5	14.5 (26)	12.2 (36)	16.9 (32)

Percentages (patient numbers) are given. The difference in distribution of AIS_Thorax_ severity between ESI and LSI groups was statistically not significant (*p* = 0.21)

Also, when checked for the distribution of additional injuries in other body regions, there were no statistical differences in the percentages of injuries with AIS ≤ 3 to the head, abdomen, extremities or pelvis (data not shown).

### Thoracic spine (TS); AIS_TS_ = 4/5

We were also able to find significant benefits for patients undergoing an operation within the first 72 h in MIP with at least severe injuries to the thoracic spine (AIS_TS_ = 4–5). They exhibited significantly shorter VT, ICUT and HT. In MIP with an AIS_TS_ of ≥4 timing of surgery had no significant influence on MOF and ARDS, but a significant reduction of sepsis rates was seen in the ESI group. The timing of surgery had no significant effect on the blood transfusions rate and GOS and there was no significant difference in mean ISS among the subgroups. The comprehensive data is shown in Table 6.

**Table 6:** Outcome of multiple injured patients with severe thoracic trauma and concomitant severe/critical thoracic spine trauma (AIS_TS_ = 4–5)

	No Surgery (NSI) *n* = 82	Early Surgery (ESI) *n* = 353	Late Surgery (LSI) *n* = 54	*P*-value (ESI versus LSI)
Mean ISS	31.7	31.6	32.5	0.19
Days on ventilator	9.2 [5]	8.0 [3]	13.7 [9]	0.038
Days on ICU	14.6 [13]	14.4 [11]	21.1 [19]	0.002
Days in hospital	39.9 [22]	32.0 [19]	37.4 [34]	< 0.001
MOF	37.3 (25)	35.4 (120)	44.7 (21)	0.22
Lung failure (ARDS)	32.8 (22)	31.0 (105)	38.3 (18)	0.31
Sepsis	7.7 (5)	10.4 (35)	21.7 (10)	0.026
Blood transfusion	15.9 (13)	26.3 (93)	20.4 (11)	0.35
GOS 2	1.4% (1)	0.9 (3)	0 (0)	0.063
GOS 3	54.1 (40)	57.4 (194)	40.7 (22)	
GOS 4	16.2 (12)	21.0 (71)	33.3 (18)	
GOS 5	14.9 (11)	15.7 (53)	24.1 (13)	

For VT, ICUT and HT the mean [median] is listed. For MOF; lung failure; sepsis, blood transfusion and GOS percentages (patient numbers) are given. The *p*-values are derived comparing the ESI and LSI group. The NSI group is listed to display the complete dataset

As mentioned above for AIS_TS_ = 3, we also checked for a possible influence of the thoracic trauma severity on the timing of surgery in the AIS_TS_ = 4–5 subgroups. Again, the distribution of AIS_Thorax_ severity was statistically not significant (data not shown).

### Lumbar spine (LS); AIS_LS_ = 3

In the subgroup of MIP with serious fractures of the lumbar spine (AIS_LS_ = 3) there were significant benefits concerning ventilation time (VT), length of stay in the intensive care unit (ICUT) or in hospital (HT), lower rates of multiple organ failure (MOF) and lung failure (ARDS) as well as blood transfusions with ESI after trauma (Table 7).

**Table 7:** Outcome of multiple injured patients with severe thoracic trauma and concomitant serious lumbar spine trauma (AIS_LS_ = 3)

	No Surgery (NSI) *n* = 114	Early Surgery (ESI) *n* = 239	Late Surgery (LSI) *n* = 126	*P*-value (ESI versus LSI)
Mean ISS	25.9	25.2	27.9	0.010
Days on ventilator	4.1 [0]	3.4 [1]	5.3 [1]	0.010
Days on ICU	8.5 [4]	8.4 [4]	12.7 [10]	< 0.001
Days in hospital	23.7 [17]	24.4 [21]	30.6 [25]	0.001
MOF	15.1 (14)	17.3 (39)	29.8 (34)	0.008
Lung failure (ARDS)	21.5 (20)	16.4 (37)	28.9 (33)	0.007
Sepsis	5.6 (5)	5.3 (12)	10.5 (12)	0.076
Blood transfusion	8.8 (10)	10.5 (25)	19.0 (24)	0.022
GOS 2	0.9 (1)	0 (0)	0.8 (1)	0.37
GOS 3	11.9 (13)	5.1 (12)	8.8 (11)	
GOS 4	20.2 (22)	25.4 (60)	27.2 (34)	
GOS 5	61.5 (67)	67.4 (159)	61.6 (77)	

For VT, ICUT and HT the mean [median] is listed. For MOF; lung failure; sepsis, blood transfusion and GOS percentages (patient numbers) are given. The *p*-values are derived comparing the ESI and LSI group. The NSI group is listed to display the complete dataset

There was a tendency towards more septic complications in the LSI group, yet these results were statistically not significant. The GOS did not differ between the subgroups with different surgical intervention times. The comprehensive data is shown in Table 7.

In contrast to the previously described AIS_TS_ groups, the AIS_LS_ = 3 group had a significantly higher mean ISS in the LSI group, as shown in Table 7. Consecutively, in the LSI group we found higher rates of additional injuries, compared to the ESI group [AIS_Abdomen_ AIS ≥ 2: ESI: 19.7% vs. LSI: 33.9%; AIS_Head_ ≥ 2: ESI: 23.5% vs. LSI: 24.2%; AIS_Extremity_ without pelvis ≥2: ESI: 44.4%; LSI: 45.2%; AIS_Pelvis_ ≥ 2: ESI: 14.5%; LSI: 23.4%].

We further analyzed whether the timing of surgery was related to the severity of thoracic trauma (AIS_Thorax_ 3–5) and found significant differences in the distribution of thoracic injury severity in the LSI group compared to ESI (*p* = 0.002, Table 8).

**Table 8:** Distribution of thoracic injury severity in multiply injured patients suffering from serious lumbar spine injury

	No Surgery (NSI) *n* = 114	Early Surgery (ESI) *n* = 239	Late Surgery (LSI) *n* = 126
AIS_Thorax_ = 3	67.5 (77)	69.5 (166)	63.5 (27)
AIS_Thorax_ = 4	23.7 (27)	25.9 (62)	21.4 (27)
AIS_Thorax_ = 5	8.8 (10)	4.6 (11)	15.1 (19)

Percentages (patient numbers) are given. The NSI group is listed to display the complete dataset. The difference in distribution of AIS_Thorax_ severity between ESI and LSI groups was statistically significant (*p* = 0.002)

### Lumbar spine (LS); AIS_LS_ = 4/5

In the subgroups of AIS_LS_ ≥ 4 there was only a tendency towards benefits of an ESI concerning MOF (*p* = 0.054). There were no significant benefits of ESI regarding VT, ICUT, HT, SR, ARDS, blood transfusion and GOS. The comprehensive data is shown in Table 9.

**Table 9:** Outcome of multiple injured patients with severe thoracic trauma and concomitant severe/critical lumbar spine trauma (AIS_LS_ = 4–5)

	No Surgery (NSI) *n* = 26	Early Surgery (ESI) *n* = 120	Late Surgery (LSI) *n* = 14	*P*-value (ESI versus LSI)
Mean ISS	38.4	39.6	43.4	0.95
Days on ventilator	3.6 [2]	4.2 [1]	7.1 [4]	0.12
Days on ICU	9.4 [7]	12.7 [9]	15.6 [14]	0.13
Days in hospital	47.2 [25]	34.1 [26]	29.5 [28]	0.46
MOF	28.0 (7)	21.9 (25)	46.2 (6)	0.054
Lung failure (ARDS)	12.0 (3)	21.1 (24)	38.5 (5)	0.16
Sepsis	0 (0)	6.1 (7)	15.4 (2)	0.22
Blood transfusion	19.2 (5)	28.6 (34)	7.7 (1)	0.088
GOS 3	26.9 (7)	36.8 (43)	35.7 (5)	0.26
GOS 4	38.5 (10)	33.3 (39)	21.4 (3)	
GOS 5	34.6 (9)	29.1 (34)	35.7 (5)	

For VT, ICUT and HT the mean [median] is listed. For MOF; lung failure; sepsis, blood transfusion and GOS percentages (patient numbers) are given. The *p*-values are derived comparing the ESI and LSI group. The NSI group is listed to display the complete dataset

There was no significant difference regarding the mean ISS between ESI und LSI (Table 9). Also, the distribution of AIS_Thorax_ severity was statistically not significant between ESI and LSI. Furthermore, there were no significant differences in additional abdominal, head, extremity and pelvic trauma (AIS ≤ 2) between the subgroups of AIS_LS_ ≥ 4 (data not shown).

## Discussion

Due to the complexity of MIP the optimal timing to stabilize spine fractures remains an ongoing issue [15, 17, 26].

One limitation in most studies dealing with multiply injured patients (ISS ≥ 16) is the heterogeneous patient collective regarding the leading injury [1, 11, 19, 27–29]. Not only do MIP exhibit different clinical courses and outcomes regarding the leading injury region, e.g. when suffering from leading head trauma in comparison to e.g. leading extremity injury [30]. Also, major injuries in different body-regions (e.g. thoracic trauma and brain injury) will have interactions and influences on the respective clinical course and outcome [31, 32]. As far as spine fractures are concerned, chest injuries (e.g. lung contusion, rib fractures, hemato- / pneumothorax) impair the respiratory system, which is additionaly affected when spine fractures exist. This has been published especially for lesions of the thoracic spine, which are often associated with high energy trauma to the chest [6–8, 33]. In summary, this is why we focused on severely injured patients with leading thoracic injuries to examine the timing of spine fixation and the influences on patients’ clinical courses in our retrospective study. By deliberately excluding more than moderate injuries to other body regions, we sought to achieve some control for the above stated confounding.

### Mortality

Kerwin et al. described risk factors for increased mortality rates, when ESI was performed in patients older than 50 years of age, with an ISS ≥ 25 or in patients with thoracic spine injury without spinal cord injury [21]. This is consistent with the literature [1, 34] and our findings. The highest rate of early and overall hospital mortality in the NSI group may be contributed to the fact that these patients might have been too instable or injured to be operated on and thus were more likely to die. This is underlined by the highest RISC II score in this subgroup. Yet, these considerations are not reflected by the mean ISS and percentage of patients with ISS ≥ 25, which is highest in the ESI group. Another explanation for the high mortality in the NSI group might be the existence of ethical statements or patient’s provisions against operations or life prolonging interventions. The higher mortality rate in the ESI group during hospital stay may be contributed to the fact that these patients were injured more seriously (higher mean ISS and rate of ISS ≥ 25) compared to the LSI group.

### Clinical course

The majority of studies and two reviews on the timing of spinal fractures in MIP showed advantages of ESI concerning neurologic outcome, VT, ICUT, HT and sepsis rate, with respect to pneumonia [1, 11, 15–19, 28, 35]. While arguments for a delayed surgical strategy are that patients are too sick to undergo early spine stabilization and the subsequent operative trauma will increase complications and worsen the patient’s condition, the possibility of an earlier mobilization, pain relief and ability for adequate positioning the patient in the ICU decreases the risk of complications like ARDS, wound infection, urinary tract infection and the development of pressure sores [17, 36, 37], thus potentially improving the clinical course of patients.

In an earlier analysis of patients from the TraumaRegister DGU® for early or delayed stabilization of spine fractures, advantages after early spine stabilization in MIP within the first 72 h were seen [1]. In comparison to ours, their collective was more heterogeneous with respect to accompanying injuries in the early and delayed intervention groups. Although both treatment groups in the study of Bliemel et al. had an ISS ≥ 16, the delayed surgical stabilization group comprised significantly more severe head, abdominal and extremity trauma patients compared to the early stabilization group (head 27.3% vs. 45.2%, abdominal 15.5 vs. 21.4%, extremity 19.8 vs. 31.2%). Additionally, they found that LSI related to MIP with life threatening injuries arriving with signs of shock, the preclinical need for catecholamines, intubation or a tube thoracostomy. Respectively, severe head injuries were the main reason for a delayed surgical treatment of spine fractures. Consistently, they described that in severely injured patients with predominantly one affected body region a trend towards an ESI prevailed. In line with that finding, the mean ISS in their ESI group was significantly lower compared to their LSI group [1]. That differs from our collective where we present severely injured patients with a mean ISS > 25 in the ESI and LSI group. This is consistent with other studies showing that an ESI is beneficial for MIP with a high ISS and thoracic fractures - both in spinal cord and non cord-injured patients [20, 22, 27, 38, 39].

Comparing our lumbar and thoracic spine groups two distinct results have to be discussed. First, like in the current literature [1, 11, 17], an ESI is especially beneficial in MIP with trauma to the thoracic spine, whereas an ESI has only partial advantages in the lumbar spine group. This is especially intriguing, since we focused on MIP with at least serious thoracic injuries and one might think that early fracture stabilization in the lumbar region should always benefit the e.g. respiratory function by earlier mobilization or more adequate patient positioning. Yet, in our study, only patients with AIS_LS_ = 3 showed significant benefits with ESI whereas AIS_LS_ = 4 + 5 did not profit from ESI. One explanation might be the mandatory neurological deficit in a lumbar spine fracture in order to be coded with AIS_LS_ = 4 or 5.

Second, the ISS in our lumbar spine group is greater than in the thoracic spine group, which might just be caused by the ISS calculation method [24], but might also be a possible explanation why the outcome parameters differ from each other.

Concerning the spinal trauma severity, we showed that early surgical stabilization of spinal fractures has especially significant benefits in MIP with relevant thoracic injuries and serious spine fractures (AIS_Spine_ = 3), regardless the affected thoracic or lumbar spine region, and in patients with at least severe injury to the thoracic spine (AIS_Spine_ ≥ 4) where advantages to almost all of our examined outcome parameters were found. This is in contrast to patients with at least severe injuries to the lumbar spine and missing effects of ESI or LSI as far as our outcome parameters are involved. To our knowledge, we are not only the first to examine ESI versus LSI in spine trauma MIP with leading relevant thoracic injuries, but also to separately scrutinize the effects of surgical timing for thoracic and lumbar spine injuries.

### Benefits of ESI and clinical implications

Patient selection for spine fixation and surgical timing seems crucial for benefits in patient survival and outcome. However, we are not able to address the question of surgical approaches (e.g. minimally invasive, solely posterior, combined anterior / posterior approaches) or duration of the operation, due to the lack of these data in our records.

Albeit, two long term prospective multicenter studies conducted by the spine working group of the German Trauma Society (“Arbeitsgemeinschaft Wirbelsäule der Deutschen Gesellschaft für Unfallchirurgie”) collected data over two distinct time periods (years 1994 to 1996 and 2002 to 2003) and shed light to the respective treatment reality in German and Austrian Level I trauma centers [40–46]. In the earlier study the majority of patients with thoracolumbar fractures underwent solely posterior stabilization, whereas the later study distinguished the surgical modality according to the level of the spinal fracture. Although their patient collective differs from ISS values (mean ISS of the subgroups was 11.4 to 14.6) they provided two interesting findings: 1) posterior instrumentation showed a lower complication rate and 2) the combined procedure had a longer operation time and a higher blood loss during surgery (152 min vs. 298 min, 650 ml vs. 959 ml) which influenced the initial coagulopathy after severe trauma [47, 48]. Concerning the operative procedures, posterior minimally invasive spine surgery, as a damage control measure, seems to be performed safely concerning neurologic outcome and operative risk [44–46].

Based on these findings and our data, we suggest a primary minimally invasive posterior stabilization in MIP with leading thoracic trauma within 72 h, especially in patients suffering from fractures of the thoracic spine with an indication for surgical stabilization and concomitant relevant thoracic injury.

### Limitations

This study is limited by its retrospective nature. Hospitals participating in the TraumaRegister DGU® are regularly audited, and sample tests are taken to ensure data quality. However, the validity of their documentation is not verified by external monitors as in prospective trials [49]. The present analysis is based on a European population and patients from different European hospitals are included in this study. Although mainly Level 1 and 2 trauma centers contribute to this database, we cannot comment on locally implemented protocols for trauma care (e.g. respiratory care, spine surgery).

Our interpretation of the causes of mortality is limited, since the actual cause of death is not recorded in the TraumaRegister DGU® database. Also, we have no data on existing patient’s provisions or ethical statements withholding therapy or implementing palliative care, thus possibly influencing the reported mortality. Additionally, we excluded patients with more than moderate injury to the head, abdomen and/or extremities (AIS ≥ 3) in our study to minimize confounding and, as a result, our findings cannot be readily transferred to severely injured patients sustaining additional major trauma to these body regions.

Another limitation is the classification of spine injuries according to the AIS, since this classification is not detailed for fracture morphology or neurologic dysfunction after spinal cord injury. Since there is no specific spine trauma classification (e.g. AO, Magerl) available in the TraumaRegister DGU® database, we do not know the circumstances of specific fracture patterns possibly influencing the decision on early or delayed spine stabilization, or formally check for surgery indicated spine fractures in the NSI group. Additionally, we are not able to comment on the performed surgical procedures (e.g. posterior / anterior approach), since these information is not available in the TraumaRegister DGU® database.

Albeit the above stated limitations, to the best of our knowledge we are the first to present comprehensive data on spinal surgery timing in multiply injured patients suffering from concomitant relevant thoracic trauma and different thoracic and lumbar spine injury severity.

## Conclusions

Multiply injured patients with at least serious thoracic trauma (AIS_Thorax_ ≥ 3) and accompanying spine trauma can significantly benefit from early spine stabilization within the first 72 h after hospital admission. Patients with injuries to the thoracic spine (AIS_TS_ = 3) or lumbar spine (AIS_LS_ = 3) significantly benefit from this early surgical intervention concerning ventilation time (AIS_LS_ = 3 only), ARDS, multiple organ failure, sepsis rate (AIS_TS_ = 3 only), length of stay in the intensive care unit and length of hospital stay. In multiple injured patients with at least severe thoracic spine trauma (AIS_TS_ ≥ 4) early surgery showed a significantly shorter ventilation time, decreased sepsis rate as well as shorter time spend in the ICU and in hospital. We suggest primary spine surgery for fracture stabilization in MIP with leading thoracic trauma, especially in patients suffering from fractures of the thoracic spine, within 72 h.

Based on our results further studies on tailored surgical strategies for multiple injured patients with leading thoracic trauma and spinal injuries are warranted.

## Data Availability

The datasets used and/or analyzed during the current study are available from the corresponding author on reasonable request.

## Abbreviations

AIS: Abbreviated injury scale
ESI: Early surgical intervention
GOS: Glasgow Outcome Scale
HT: Length of hospital stay
ICU: Intensive care unit
ICUT: Length of ICU stay
IMC: Intermediate care unit
ISS: Injury severity score
LS: Lumbar spine
LSI: Late surgical intervention
MIP: Multiply injured patients
MOF: Multiple organ failure
NSI: No surgical intervention
RR_sys_: Systolic blood pressure
SR: Sepsis rate
TS: Thoracic spine
VT: Ventilation time
